# A semi-Markov model for stroke with piecewise-constant hazards in the presence of left, right and interval censoring

**DOI:** 10.1002/sim.5534

**Published:** 2012-08-18

**Authors:** Venediktos Kapetanakis, Fiona E Matthews, Ardo Hout

**Affiliations:** a National Heart Forum LondonU.K.; b MRC Biostatistics Unit Institute of Public Health CambridgeU.K.; c Department of Statistical Science University College London LondonU.K.

**Keywords:** censored data, semi-Markov model, multi-state modelling, piecewise-constant hazards, EM algorithm, stroke

## Abstract

This paper presents a parametric method of fitting semi-Markov models with piecewise-constant hazards in the presence of left, right and interval censoring. We investigate transition intensities in a three-state illness–death model with no recovery. We relax the Markov assumption by adjusting the intensity for the transition from state 2 (illness) to state 3 (death) for the time spent in state 2 through a time-varying covariate. This involves the exact time of the transition from state 1 (healthy) to state 2. When the data are subject to left or interval censoring, this time is unknown. In the estimation of the likelihood, we take into account interval censoring by integrating out all possible times for the transition from state 1 to state 2. For left censoring, we use an Expectation–Maximisation inspired algorithm. A simulation study reflects the performance of the method. The proposed combination of statistical procedures provides great flexibility. We illustrate the method in an application by using data on stroke onset for the older population from the UK Medical Research Council Cognitive Function and Ageing Study. Copyright © 2012 John Wiley & Sons, Ltd.

## 1 Introduction

Stroke is the rapidly developing loss of brain function due to a disorder in the blood supply to the brain. It can cause serious complications that may lead to death. Stroke is the third largest cause of death in the UK and the USA 1,2. Non-fatal stroke may cause serious complications including permanent neurological damage and adult disability.

Multi-state modelling is a method of analysing longitudinal data when the observed outcome is a categorical variable. In medical research, multi-state models are often used to model the development or progression of a disease, where the different levels of the disease can be seen as the states of the model. This approach enables the investigation of ageing in the older population by jointly modelling the rate of having a non-fatal stroke or dying on healthy individuals and the rate of dying after having a non-fatal stroke. Multi-state models have been used in a wide range of applications including AIDS 3, liver cirrhosis 4, cognitive impairment 5, coronary heart disease 6, stroke 7 and various types of cancer 8,9. 10Putter *et al.* 2007 have published a concise introduction to multi-state modelling.

Norris 1997^11^ has discussed the theory of stochastic processes and Markov chains. Fitting multi-state models involves various assumptions. A common hypothesis is that the data satisfy the first-order time-homogeneous Markov property. According to this assumption, the transition to the next state depends only on the current state. This means that any previous history of the process can be ignored. Although this assumption simplifies statistical modelling, it may often be inappropriate and lead to incorrect conclusions. A number of extensions to the theory have been proposed including the incorporation of history in the underlying stochastic process. Weiss and Zelen 12 first proposed a semi-Markov model for clinical trials. In semi-Markov models, the transition to the next state depends not only on the current state but also on the time spent in the current state. This involves the exact transition time from one state to the other, which in many applications is unknown. In 1999, Commenges introduced the terminology of a partial Markov model 13. In partial Markov models, the transition to the next state depends not only on the current state but also on a multivariate explanatory process that can be predicted at the current state. This enables the inclusion of explanatory covariates in multi-state modelling. Faddy 14 applied originally a model with piecewise-constant transition intensities, which enables intensities to depend on time-varying covariates. Van den Hout and Matthews^15^ have also discussed a piecewise-constant approach for the effect estimation of explanatory variables in multi-state modelling.

Longitudinal studies, as opposed to cross-sectional studies, involve repeated observations on the same individuals over time. In such studies, researchers often recruit individuals over a range of ages at which some participants may have already developed and progressed through the different study endpoints. Longitudinal data are usually collected by monitoring individuals at prespecified times over the period of an observational study. Thus, the value of monitored variables is known at a discrete set of times, only. The case where the exact value of a variable is unknown and only partial information is available is referred to as censoring 16. There are three types of censoring, namely left, right and interval censoring. In left and right censoring, the value of a variable is known to lie below and above a certain value, respectively. In interval censoring, the value of a variable is known to lie within an interval with known limits. Methods for handling right-censored data have been discussed in a number of statistical textbooks 17,18 and are widely implemented in medical research. However, methods for adjusting for left censoring are less frequently employed in longitudinal studies 19. Ignoring the presence of left censoring when estimating the underlying stochastic process that explains the data observed, may cause substantial bias 19. Cain *et al.* have shown that including individuals whose data are subject to left censoring (by collecting all necessary information at the time of recruitment) rather than excluding them from the analysis reduces bias significantly 2011. A notion similar to left censoring is that of left truncation. However, left truncation is to be distinguished from left censoring. A left-truncated distribution is one formed from another distribution by cutting off and ignoring the part lying to the left of a fixed variable value 20. A left-truncated sample is likewise obtained by ignoring all values smaller than a fixed value 20. Left truncation may occur in longitudinal studies when individuals who have already developed and progressed through the different study endpoints before the beginning of the study are not included in the study. A reason for an individual not to be included in the study is the event of death before the initiation of the study. In 1986, Kay 21 introduced a method that dealt with the problem of right censoring and also handled the case where the time of death is known precisely. Foucher *et al.* have investigated ways to fit multi-state models in the presence of left, right and interval censoring by using a generalised Weibull distribution for the waiting times of the underlying process 22. Interval censoring has often been dealt with by integration 6. In 1993, Lindsey and Ryan 8 presented another approach for adjusting for interval censoring based on the Expectation–Maximisation (EM) algorithm.

This paper presents a method to incorporate history in the underlying process in the presence of left truncation and left, right and interval censoring. The proposed model combines properties of semi-Markov models and partial Markov models. We handle interval censoring by integration and adjust for left censoring by using an EM-inspired algorithm 23. We bypass left truncation by analysing data only over the period of follow-up although, for the adjustment for left censoring, assumptions about the process before baseline need to be made. We illustrate the method in an application by using data from the UK Medical Research Council Cognitive Function and Ageing Study (MRC CFAS). The objective was to investigate ageing in the older population by modelling the transition intensities in a three-state model that comprises the states ‘healthy’ (state 1), ‘history of stroke’ (state 2) and ‘death’ (state 3) and to investigate how time after an individual has a stroke affects the rate of dying. Statistical inference about ageing is feasible only for the older population because the study includes individuals in their 65th year and above. Survival after having a stroke has been discussed in several articles 24,1. These articles assist the understanding of the mechanisms and the difficulties that exist in the particular data set that is used in the application and enable the validation of the results of the proposed method.

Section 2 presents the available data of the MRC CFAS. Section 3 presents the statistical model and the method to include time-varying explanatory covariates in the presence of right and interval censoring. We discuss handling left censoring in Section 7. A simulation study in Section 8 shows how assumptions about the process before baseline affect the performance of the method. Section 9 illustrates the method on the MRC CFAS data and investigates model fit graphically. Finally, Section 0010 is the discussion.

## 2 Data

The MRC CFAS is a large scale multi-centre longitudinal study conducted in the UK 25. The study was launched in the late 1980s to explore dementia and cognitive decline by using a representative sample of 13 004 people in the older population. The data have also been used to investigate other disorders such as depression 26 and physical disability 27 and to look at healthy active life expectancy 15. To date, over 46 000 interviews with participants have been completed. More information on the design of this study is available online (http://www.cfas.ac.uk).

**Figure 1:**
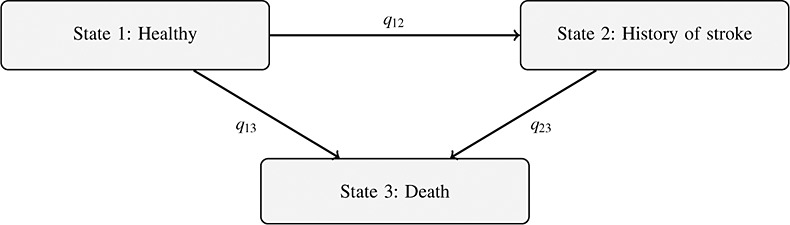
Three-state model for data from the Medical Research Council Cognitive Function and Ageing Study.

The objective in this paper was to investigate ageing in the older population by modelling the transition intensities in a three-state model that comprises the states ‘healthy’ (state 1), ‘history of stroke’ (state 2) and ‘death’ (state 3). Figure 1 illustrates the multi-state model. Of interest is how time after an individual has a stroke affects the rate of dying.

We analyse a subset of the MRC CFAS data, that is, data of the Newcastle centre only. We denote this data set as the MRC CFAS throughout this work. This subset includes data of 2316 individuals in their 65th year and above, interviewed during the period from 1991 to 2003. These individuals had up to nine interviews where they were asked whether they had had a stroke since they were last seen, and age at interviews was recorded. Exact dates of death are available even after the end of follow-up. At baseline, history of stroke up to that time was investigated, and individual data for age (*A*), gender (*G*; 0 for women and 1 for men), years of education (*E*; 0 for less than 10 years and 1 for 10 years or more) and smoking status at age 60 years (*S*; 0 for non-smoker or ex-smoker and 1 for current smoker) were collected. Defining smoking in this way reduces a bias from giving up due to ill health. Smoking habits rarely change after age 60 years. According to the annual report for smoking-related behaviour and attitudes in 2005 28, smokers over the age 65 years are the least likely to want to stop smoking, and those who want to give up are more likely to have quit before the age of 65 years.

**Figure 2:**
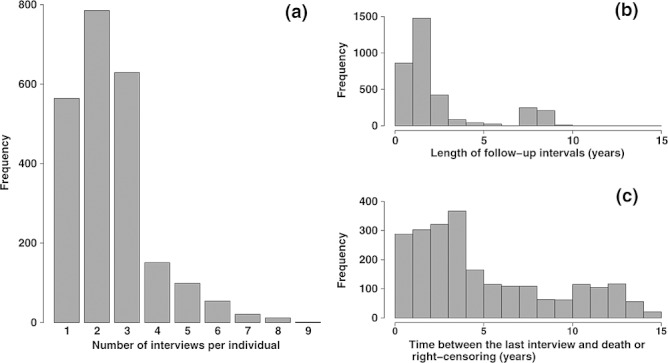
Descriptive statistics of (a) the number of interviews per individual, (b) the time between interviews and (c) the time between the last interview and either death or censoring.

Both the number of interviews and the time between interviews varied among individuals. Figure 2(a) and (b) show the number of interviews per individual and the distribution of the length of follow-up intervals, respectively. The median length of follow-up intervals was 2 years, and the median number of interviews was 2. Figure 2(c) illustrates the distribution of the time between the last interview and the time of either death or right censoring. Table 1 shows the frequencies of pairs of consecutive states in the data. For each state *i* and *j* and over all individuals, these frequencies correspond to the number of times an individual had an observation in state *i* followed by an observation in state *j*. Owing to the definitions of the states, there were no transitions from state 2 to state 1.

**Table 1:** Frequencies of pairs of consecutive states, corresponding to the number of times an individual had an observation in state *i* followed by an observation in state *j*, as observed in the MRC CFAS data.

	To (state *j*)					Total
Healthy	History of stroke	Death	Censored
From (state *i*)					
Healthy	2964	113	1328	710	5115
History of stroke	0	303	223	55	581
Total	2964	416	1551	765	5696

**Figure 3:**
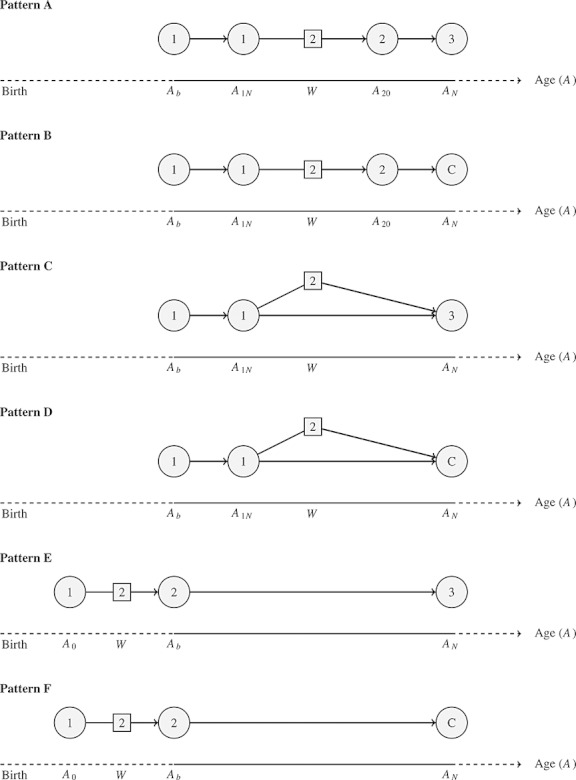
Data patterns with regard to possible transitions between the three states. C denotes censoring. *A*_0_ is age at which all individuals are assumed to be healthy, *A*_*b*_ is age at baseline, *A*_1*N*_ is age at the last time an individual is observed in state 1, *A*_20_ is age at the first time an individual is observed in state 2, *A*_*N*_ is age at the end of the follow-up, and *W* is age at the time of transition from state 1 to state 2.

In the MRC CFAS longitudinal study, there are a number of potentially observed patterns of follow-up for each individual. For example, if at the beginning of the study an individual is healthy, then he or she can either have a stroke in the coming years and die or be still alive when the study ends, or *not* have a stroke and either die before the end of the study or be right censored. Likewise, if at the beginning of the study an individual is reported to have had a stroke, then he or she may remain alive or die before the end of the study. We depict these various patterns graphically in Figure 3 and label them as separate patterns A–F. In patterns A, B, E and F, a transition from state 1 to state 2 is known to have happened. For patterns C and D, however, the presence of censoring makes it impossible to know whether such a transition has taken place. Therefore, two scenarios are possible. An individual may have moved to state 2 and never been recorded in this state owing to censoring or may have remained in state 1 until he or she died or the state was right censored.

In the data, 2151 individuals were observed in state 1 at baseline, whereas 165 had a stroke before the beginning of the study. Individuals who had a stroke before the initiation of the study were asked at their first interview to report the time of their first stroke. Self-reported data are often subject to measurement error due to digit preference, that is, the tendency to round outcomes to pleasing digits 29, and should be treated with caution. Moreover, in most longitudinal studies, information about the measured endpoints prior to baseline is rarely available. For this reason, we have developed in this paper a method that does not need this information and have not use self-reported data regarding the time of first stroke. Hence, the way the proposed method handles all types of censoring makes the method applicable to most longitudinal studies.

The median age of individuals at baseline was 74 years. This was imposed by the study design, according to which individuals over their 75th year were over-sampled to achieve equal numbers with individuals aged 65–74 years at baseline. By the study design, every individual was followed up approximately every 2 years. The time of death is known exactly. Because the exact time of transition from state 1 to state 2 is unknown, the data are subject to left, right and interval censoring. It is possible that transitions from state 1 to state 2 may have occurred and not have been observed before death or right censoring at the end of follow-up. Transitions from state 1 to state 2 that take place before the beginning of the study are left censored if individuals are enrolled in the study or left truncated if they are not. A reason for an individual not to be enrolled is the event of death before baseline.

To include the individuals who were observed in state 2 at the beginning of the study, the estimation of the exact age of onset of state 2 is necessary. This estimation involves assumptions with regard to the age at which these individuals were healthy in the past. Using data published in ‘Key health statistics from general practice’ reports of the Office for National Statistics 30, we estimated that 90% of individuals who have a stroke before the age of 76 years (the median age at baseline for individuals who had a stroke before the beginning of the study) have the stroke after the age of 40 years. For these individuals, the probability of having the stroke within the age span 35–44 and 45–55 years is 5.06% and 12.6%, respectively. In the estimation of these figures, we ignored possible cohort effects owing to unavailability of data. Nevertheless, we expect the true estimates to be of similar magnitude. Therefore, when modelling stroke for individuals who were observed in state 2 at baseline, a realistic assumption with regard to the age at which these individuals can be assumed to have been healthy in the past is to assume that they were healthy at the age of 40 years.

To overcome the difficulties imposed by the study design and the presence of censoring, we used *age*, *A*, as the time scale. Age is also the natural time scale for processes in the older population. We introduce the following notation:

*A*_0_:Age before the beginning of the study at which all individuals are assumed to have been healthy.

*A*_*b*_:Age at baseline.

*A*_1*N*_:Age at the last time an individual is observed in state 1.

*A*_20_:Age at the first time an individual is observed in state 2.

*A*_*N*_:Age at the end of the follow-up.

*W*:Age at the time of transition from state 1 to state 2.

## 3 Method

We model the semi-Markov process via regression equations for transition intensities and fit the three-state model illustrated in Figure 1, adjusting all transition intensities for a number of possible confounders. We introduce history in the underlying stochastic process by fitting a semi-Markov model where the waiting time in state 2 is used as a time-varying covariate.

### 3.1 The regression model

Let *Y* (*A*) ∈ {1,2,3} denote an individual's state at age *A*, and let *q*_*ij*_ be the intensity for the transition from state *i* to state *j*, where (*i*,*j*) ∈ {(1,2),(1,3),(2,3)}. We model the transition intensities as follows: 

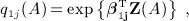
(1)

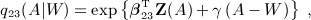
(2)
where *j* = 2 or 3; **Z**(*A*) = (1,*A*,*X*_1_,*X*_2_, …, *X*_*r*_)^T^ is a vector including age, *A*, and *r* explanatory variables, *X*_1_,*X*_2_, …, *X*_*r*_; 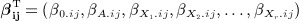 is the vector of corresponding regression coefficients for a transition from state *i* to state *j*; and *W* is age at the time of transition from state 1 to state 2. The parameter *γ* is the regression coefficient corresponding to the time spent in state 2, *A* − *W*. Without loss of generality, we assume that the explanatory variables *X*_1_,*X*_2_, …, *X*_*r*_ may vary with age, *A*. Throughout this work, the vector (1,*A*,*X*_1_,*X*_2_, …, *X*_*r*_)^T^, evaluated when an individual is *A* years old, is denoted by **Z**(*A*).

For simplicity, we consider the restricted model when **Z**(*A*) = (1,*A*)^T^. The transition intensities *q*_1*j*_(*A*) for *j* ∈ {2,3}can be expressed as follows: 

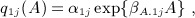

where 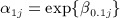. Thus, the baseline intensity function for a transition from state 1 to state *j* ∈ {2,3}comes from a Gompertz distribution with age as the time scale. Similarly, transition intensity *q*_23_(*A* | *W*) can be expressed as follows: 

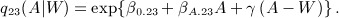

If we let *T*_2_ = *A* − *W*, then transition intensity *q*_23_ takes the following form: 

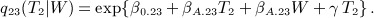

When an individual moves to state 2, *W* remains constant thereafter, and *q*_23_ can be expressed as follows: 

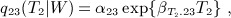
(3)
where 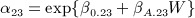 and 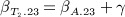. This equation shows that the baseline intensity function for a transition from state 2 to state 3 comes from a Gompertz distribution with time spent in state 2 as the time scale.

The Gompertz distribution is suitable for modelling data with monotonic hazard rates that either increase or decrease exponentially with time. In Equation 3, when 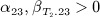, the hazard function increases with time, and survival tends to 0 as *T*_2_ tends to infinity. When 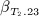 is zero, the hazard function is equal to *α*_23_ for all *T*_2_, so the model reduces to an exponential. A negative 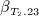 would imply that the hazard function decreases with time. However, when *α*_23_ *≤*0 or 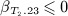, the cumulative distribution function is improper. In particular, when *α*_23_ > 0 and 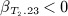, survival tends to 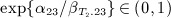 as *T*_2_ goes to infinity. This implies that 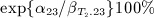 of individuals never move to state 3. Similarly, in Equation 2, when − *β*_*A*.23_ < *γ* < 0, an individual's mortality after having a stroke increases with age, but this increase becomes smaller with time after the stroke. However, when *γ* < − *β*_*A*.23_ < 0 (i.e. 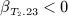), the model implies that there is a non-zero probability of never failing (living forever). That is, there is always a non-zero hazard rate, yet it decreases exponentially.

Some authors of recent survival analysis textbooks would restrict 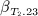 to be strictly positive so that the survivor function always goes to zero as *T*_2_ tends to infinity 31. Although this may be a desirable mathematical property, the more traditional approach that also standard statistical software take is that of not restricting 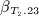 32. The main reason for this is that, in survival studies, individuals are not monitored forever. There is a date when the study ends, and in many applications in medical research, an exponentially decreasing hazard rate is clinically appealing 32.

## 3.2 A piecewise-constant hazards model

**Figure 4:**
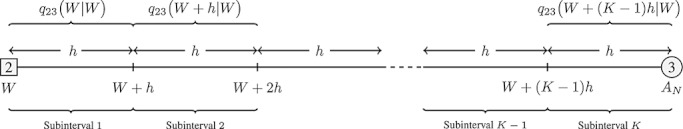
Illustration of the splitting process of the time scale of age, which allows for piecewise-constant intensities for a transition from state 2 to state 3. Transition intensities are evaluated at the left limit of each subinterval. *W* is age at the exact time of transition from state 1 to state 2. *A*_*N*_ is age at the end of follow-up.

The transition intensities are functions that vary with age, and this should be taken into account in the estimation of the likelihood contributions. In particular, all the transition intensities should be adjusted for age, *A*, and explanatory variables, *X*_1_,*X*_2_, …, *X*_*r*_, which may vary with age. Furthermore, the transition intensity *q*_23_ should be adjusted for the time spent in state 2, *A* − *W*, in a time-varying way. Let [*A*_*L*_,*A*_*U*_] be any age interval with lower and upper age limits *A*_*L*_ and *A*_*U*_, respectively. A piecewise-constant approach for the transition intensities involves splitting [*A*_*L*_,*A*_*U*_] into small pieces, within which the transition intensities are assumed to be constant. More precisely, a resolution, *h*, is specified; and starting from *A*_*L*_, interval [*A*_*L*_,*A*_*U*_] is split into as many as possible subintervals of length *h*. Without loss of generality, we assume that [*A*_*L*_,*A*_*U*_] can be split in exactly *K* such subintervals. In every subinterval *k*, *k* = 1, …, *K*, the transition intensities are evaluated at the left subinterval limit. Figure 4 illustrates the piecewise-constant approach for a transition from state 2 to state 3. In this case, *A*_*L*_ = *W* and *A*_*U*_ = *A*_*N*_. As an example, at subinterval 2, we evaluate *q*_23_ at age *W* + *h*. We assume the time spent in state 2 throughout subinterval 2 to be (*W* + *h*) − *W* = *h*.

### 3.3 Likelihood contributions

To fit the model defined by Equations 1 and 2, age at the exact time of transition from state 1 to state 2, *W*, is needed. However, *W* is unknown for all patterns. For data that follow patterns A–D, this problem is solved by integrating out all possible values of *W* in the calculation of the likelihood contribution of every individual. Equations 7 and 8 give the individuals' contribution to the likelihood for patterns A and D, respectively. These likelihood contributions are conditional on individuals' state at baseline, *Y* (*A*_*b*_). 

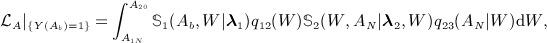
(4)

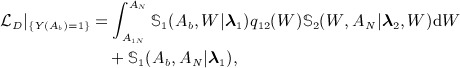
(5)
where 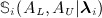 is the probability that an individual remains in state *i* throughout age interval [*A*_*L*_,*A*_*U*_] when the escape rate from state *i* is ***λ***_*i*_ for *i* ∈ {1,2}. Under the assumption of piecewise-constant transition intensities, it follows that ***λ***_*i*_ is piecewise constant over the same subintervals as the transition intensities and that the probability of remaining in state *i* at a specific subinterval *k* follows an exponential distribution with parameter *λ*_*i*.*k*_. Hence, for every age interval [*A*_*L*_,*A*_*U*_], it follows that 

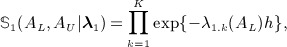
(6)

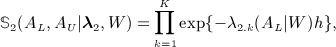
(7)
where 

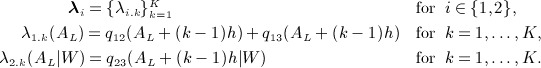


The rationale behind Equations 7 and 8 becomes clear by looking at Figure 3. In pattern A, every individual is healthy at the beginning of the study, moves to state 2 at some point and then dies. The time of the transition to state 2 is unknown. Under the assumption that this transition takes place when the individual is *W* years old, the likelihood contribution of this individual is given by the integrand of the integral in Equation 7. That is the probability that the individual remains in state 1 for time *W* − *A*_*b*_ following a distribution with piecewise-constant rate *q*_12_ + *q*_13_, then moves to state 2 with an instant transition rate *q*_12_, then remains in state 2 for time *A*_*N*_ − *W* following a distribution with piecewise-constant rate *q*_23_, and finally moves to state 3. However, the exact value of *W* is unknown. We obtain the likelihood contribution by integrating out all possible values for *W* in (*A*_1*N*_,*A*_20_).

In pattern D, two different things can happen, and the likelihood contribution of each individual consists of two terms. The first term corresponds to the case where a transition to state 2 *does* take place. In this case, the likelihood contribution is given by a similar integral as in pattern A. The difference is the right censoring at the end of the follow-up. In pattern D, we include the probability of remaining in state 2 for at least time *A*_*N*_ − *W*, but there is no multiplication with the rate of transition to state 3 at the end of the follow-up. The second term in Equation 8 corresponds to the case where a transition to state 2 *does not* happen. The likelihood contribution is then given by the probability of remaining in state 1 throughout interval [*A*_*b*_,*A*_*N*_].

For data patterns B and C, we calculate the likelihood contributions in a similar way. For patterns E and F, where the transition from state 1 to state 2 takes place before baseline, we estimate the time of onset of state 2 as described in Section 7. Once this estimate is obtained, the model for these patterns is fully specified. Hence, for individuals in patterns E and F, their contribution to the likelihood is 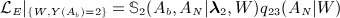 and 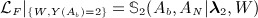, respectively, where 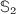 is given by Equation 10. These likelihood contributions are conditional on individuals' state at baseline, *Y* (*A*_*b*_) and *W*. We calculate the full likelihood, 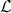, by multiplying all the individual likelihood contributions.

## 4 Handling left censoring

When the data are subject to left censoring (patterns E and F), there is no left age limit at which the individual is known to have been healthy. However, we assume that there is some age, *A*_0_, at which all individuals in the data were healthy. Figure 3 illustrates the assumption. Let 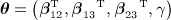 be the parameter vector of the regression model defined by Equations 1 and 2. We use a method inspired by the EM algorithm 23 to handle left censoring, as follows:

1. We fit the model defined by Equations 1 and 2 for individuals in patterns A–D to obtain maximum likelihood parameter ***θ***^0^.
2. We use parameter ***θ***^0^ to find the expected age of transition from state 1 to state 2, *W*, for individuals in patterns E and F. An estimate of the time spent in state 2 for individuals in patterns E and F at baseline is *A*_*b*_ − *W*.
3. We refit the model defined by Equations 1 and 2, including individuals from all patterns, by using *A*_*b*_ − *W* obtained from the previous step as the time spent in state 2 at baseline, to obtain new maximum likelihood parameter ***θ***^1^.
4. We repeat steps 2 and 3, creating a sequence of parameter vectors {***θ***^0^,***θ***^1^, … }, until convergence of both the parameter vector and the maximum of the likelihood.
5. After obtaining the converged maximum likelihood parameters of ***θ***, we find confidence intervals for the parameters by using the non-parametric bootstrap method. We obtain each bootstrap sample by weighting all sampling units of the available data with equal weight, and by randomly taking a sample of equal size from the available data, with replacement. In longitudinal studies where an individual may have multiple records, every individual is one sampling unit. The resampling scheme is stratified by the following strata: (i) patterns A–D and (ii) patterns E and F. Stratification preserves the same proportion of left-truncated and left-censored data in all bootstrap samples and eliminates a potential source of bias. We estimate the variance of the mean by using the studentised bootstrap statistic and the non-parametric delta method, as described by Davison and Hinkley 33.

We compute the expected age at the time of transition from state 1 to state 2 in step 2 by splitting interval [*A*_0_,*A*_*b*_] following the piecewise-constant approach described in Section 3.2 and by finding the probability of transition in each subinterval conditioning on the fact that a transition from state 1 to state 2 takes place within [*A*_0_,*A*_*b*_]. Without loss of generality, let us assume that [*A*_0_,*A*_*b*_] can be split into exactly *K* such subintervals. For each *k* = 1, …, *K*, let *P*_*k*_ be the probability that an individual moves to state 2 within subinterval *k*, conditional on that he or she is healthy at age *A*_0_, and is found in state 2 at baseline, that is, 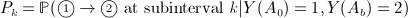, where *Y* (*A*) is the underlying multi-state process of an individual at the time point where he or she is *A* years old. Using Bayes' theorem, we can obtain *P*_*k*_ as 

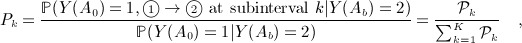
(8)
where 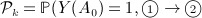 at subinterval *k* | *Y* (*A*_*b*_) = 2), which is given by the following formulas: 

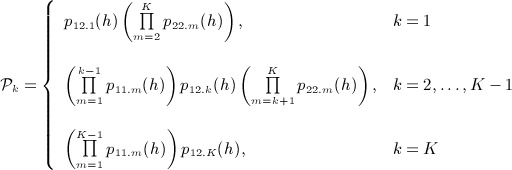
(9)
where *p*_12.*k*_(*h*) is the probability of transition from state 1 to state 2 within time *h* at subinterval *k* and *p*_*ii*.*k*_(*h*) is the probability of remaining in state *i* ∈ {1,2}for time *h* at subinterval *k*, for *k* = 1, …, *K*. The probabilities *P*_*k*_ in Equation 1 simplify the problem of finding the expected age at the time of transition from state 1 to state 2 within [*A*_0_,*A*_*b*_] by reducing it to the calculation of the mean of a multinomial distribution with *K* possible outcomes with corresponding probabilities *P*_1_, …, *P*_*K*_. The expected subinterval of the transition from state 1 to state 2 is therefore subinterval k*, where 

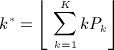
(10)
and ⌊ ⋅ ⌋is the floor function, which maps a real number to the largest preceding integer. The expected time of transition within subinterval k*, conditional on that a transition has taken place within subinterval k*, is 

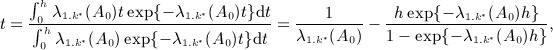
(11)
where 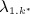 is the escape rate from state 1, evaluated at the beginning of subinterval k*. Hence, the age at which an individual in pattern E and F is expected to move from state 1 to state 2 is 

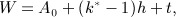
(12)
where k* and *t* are given by Equations 14 and 15, respectively.

## 5 Simulation study

Assuming that *A*_0_ = 40 for the 165 individuals in patterns E and F in the MRC CFAS data implies that for these individuals, *A*_*b*_ − *A*_0_ is 36 years on average. This period is three times longer than the time of study follow-up, *A*_*N*_ − *A*_*b*_, which has a maximum of 12 years. To examine whether this assumption influences the performance of the proposed method, we carried out a simulation study with two investigated scenarios (Scenarios I and II). The differences between the two simulation scenarios were the age at which everyone was assumed to have been healthy before baseline, *A*_0_, and the distribution of age at baseline. In Scenario I, we assumed *A*_0_ to be 60 years and we simulated age at baseline from a normal distribution with mean 65 years and standard deviation 2 years, left truncated at the age of 64 years. We chose this combination so as to reflect moderate left censoring, and this implied that the range of *A*_*b*_ − *A*_0_ was 4 to 12 years with an average of 6 years. In Scenario II, we assumed *A*_0_ to be 40 years and we simulated age at baseline from a normal distribution with mean 75 years and standard deviation 6.5 years, left truncated at the age of 64 years. This distribution had equal mean and range as the observed age at baseline in the MRC CFAS data, although age at baseline in the MRC CFAS was bimodal by design.

In both simulation scenarios, we simulated data according to Equations 1 and 2, adjusting all transition intensities for age, *A*. We additionally adjusted the transition intensity *q*_23_ for the time spent in state 2. We included no other covariates in the simulation study. Although time-varying covariates have not been included in the simulation study, such covariates can be included after postulating a model for their trajectories with age.

Having the intention to select appropriate values for the model coefficients in the simulation study, we chose the values of *β*_0.12_,*β*_0.13_,*β*_0.23_,*β*_*A*.12_,*β*_*A*.13_ and *β*_*A*.23_ after fitting a three-state multi-state model to the MRC CFAS data with age as the only covariate. We fitted the multi-state model by using the msm package 34 in R following a piecewise-constant approach, which allowed transition intensities to change after every observation point. We investigated several values for *γ*. When simulating individuals' trajectories, positive values of *γ* made individuals move to state 3 very quickly after a simulated transition to state 2. We avoided this by choosing *γ* to be negative. Table 2 shows the selected values of model coefficients for both simulation scenarios. As discussed in Section 3.1, choosing *γ* to be − 0.11 and *β*_*A*.23_ to be 0.052 implies that a maximum of 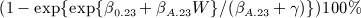 of individuals die after having a stroke. Thus, a maximum of 74.3% of individuals with simulated stroke at the age of 65 years will eventually move to state 3. This makes the survival function for transitions from state 2 to state 3 improper. Although the selected value of *γ* involves an unrealistic long-term survival, this choice facilitates the simulation study as it leads to simulated trajectories with non-negligible time spent in state 2. The choice of *γ* should not affect the findings of the simulation study.

**Table 2:** In both simulation scenarios (I,II): analysed data patterns = A − F, individuals at baseline = 1500, number of simulated data sets = 1000, time between interviews = 24 months, time of follow-up = 12 years. In Scenario I: *A*_0_ = 60, *A*_*b*_ ∼ *N*(65,2), left truncated at the age of 64 years. In Scenario II: *A*_0_ = 40, *A*_*b*_ ∼ *N*(74,6.5), left truncated at the age of 64 years.

Regression coefficient	True value	Scenario I					Scenario II							
Mean estimate	Percentage bias (%)	RMSE	Mean estimate	Percentage bias (%)	RMSE
*β*_0.12_	− 8.780	− 8.962	2.07	1.818	− 8.631	1.70	1.057
*β*_0.13_	− 10.310	− 10.365	0.53	1.020	− 10.240	0.68	0.638
*β*_0.23_	− 5.920	− 5.888	0.53	2.212	− 5.522	6.73	1.033
*β*_*A*.12_	0.065	0.067	2.87	0.025	0.062	4.03	0.014
*β*_*A*.13_	0.093	0.094	0.87	0.014	0.092	0.83	0.008
*β*_*A*.23_	0.052	0.050	3.10	0.031	0.046	11.98	0.013
*γ*	− 0.110	− 0.102	7.71	0.034	− 0.087	21.14	0.026

We simulated age at baseline, *A*_*b*_, for all individuals. All simulated data sets included 1500 individuals at baseline. To obtain this sample size, we simulated trajectories for 2500 individuals starting from age *A*_0_. We excluded from the analysis individuals who moved to state 3 before the simulated age at baseline, treating their data as left truncated. In all simulations, more than 1500 individuals were alive at age *A*_*b*_. However, all analyses included 1500 individuals in order to have the same sample size in all simulated data sets. When simulating the trajectories of individuals, we chose the resolution of the piecewise-constant approach so that the transition intensities changed with age every 0.25 year (3 months). For both simulation scenarios, the length of follow-up was 12 years, and the length of follow-up intervals was 2 years.

In both simulation scenarios, we fitted the model defined by Equations 1 and 2 on 1000 simulated data sets. When fitting the model, we calculated the integrals for the calculation of the likelihood by using the composite Simpson's rule for numerical integration 35, with resolution equal to 0.05 year (18 days). The average percentage of individuals with left-truncated data was significantly greater in simulation Scenario II (36.8%) as compared with Scenario I (7.8%) because individuals' simulated entry to the study was at a more advanced age. Moreover, the percentage of individuals observed in state 2 at baseline was 20.3% and 4.9% for simulation Scenarios II and I, respectively. Table 2 shows the results of Scenarios I and II. Mean estimates in both scenarios converged within 250 simulations. In Scenario I, the estimates of all regression coefficients were very close to their true values. Percentage bias remained below 5% for all coefficients except for the one which corresponds to the time spent in state 2 (percentage bias for *γ* = 7.71%). Nevertheless, the actual bias for *γ* was very small ( bias = 0.008). In Scenario II, the coefficients corresponding to intensities *q*_12_ and *q*_13_ were close to their true values. However, we estimated the coefficients corresponding to *q*_23_ with more than 6% bias. In particular, the percentage bias for *γ* was more than 21%. The presence of extensive left censoring and left truncation had greater impact on the coefficients, which model the rate of escape from state 2. There are two reasons for this. The first reason is the fact that left censoring is handled by the EM-inspired algorithm. When *A*_*b*_ − *A*_0_ is large, the difference between the expected age of onset of state 2 in the expectation step of the EM-inspired algorithm and the true age of onset of state 2 within the interval [*A*_0_,*A*_*b*_] may be large. When *A*_*b*_ − *A*_0_ is small, the estimation of the true age of onset of state 2 becomes more accurate. The second reason is that the likelihood contributions of individuals in patterns E and F include only *q*_23_ and not *q*_12_ or *q*_13_. Because left censoring appears only in patterns E and F, it affects mostly intensity *q*_23_. Intensities *q*_12_ and *q*_13_ are affected by left censoring to a lesser extent.

The square root of the mean squared error (RMSE) of the regression coefficients, although similar, was consistently smaller in Scenario II as compared with Scenario I. Although this is counter-intuitive, it can be explained. For the chosen true values of the regression coefficients in these simulations, the percentage of individuals observed in state 2 at baseline was more than four times greater in Scenario II (20.3%) than that in Scenario I (4.9%). When the percentage of individuals in patterns E and F is larger, there is more information for the estimation of *q*_23_. Because the transition intensities are jointly modelled, this affects the precision of all parameter estimates. This is why all RMSE estimates were smaller in Scenario II as compared with Scenario I. However, it is important to make clear that simulation Scenarios I and II are not comparable because both, *A*_0_ and 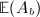, were chosen differently. Scenario I has the capacity to show that the method works well in the presence of moderate left censoring and left truncation, where *A*_*b*_ − *A*_0_ is small as compared with *A*_*N*_ − *A*_0_. In Scenario II, both, *A*_0_ and 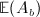, were chosen to be smaller and larger than in Scenario I, respectively, to investigate the performance of the method in the case of severe left censoring and truncation. Smaller simulation studies (not presented in this paper) where the distribution of *A*_*b*_ was fixed allowing only *A*_0_ to vary confirmed that the proposed method works well when *A*_*b*_ − *A*_0_ is reduced, independently of whether this is achieved by increasing *A*_0_, reducing *A*_*b*_ or both.

Apart from the value of *A*_0_ and the distribution of *A*_*b*_, the results of the simulation scenarios also depend on the chosen values of the regression coefficients that were used to simulate the data. These coefficients affect the extent of left censoring and left truncation. Although the distribution of *A*_*b*_ had equal mean and range as the observed age at baseline in the MRC CFAS data, the percentage of individuals observed in state 2 at baseline was 20.3% in Scenario II and 7.1% in the MRC CFAS. Of those who had had a stroke and reported their history in the MRC CFAS, 98% expressed their first stroke after the age of 40 years. Moreover, 59% and 79% of these individuals had had their first stroke within 5 and 10 years of baseline interview, respectively. On the contrary, in Scenario II, the relative figures for 5 and 10 years of baseline interview were 29% and 48%, respectively, therefore showing that the data in the MRC CFAS are subject to less severe left censoring than the data simulated in Scenario II. This, and the fact that the percentage of left-censored data in the MRC CFAS is comparable with that of Scenario I (7.1% and 4.9%, respectively), indicate that the method is applicable to the MRC CFAS data. To investigate the effect of length of follow-up intervals, length of follow-up and sample size, we carried out additional simulations based on a range of different scenarios. The results (not presented) confirmed that bias is reduced by more frequent interviews and longer follow-up and showed that the proposed method performs equally well with 500, 1000 and 1500 individuals.

## 6 Application: The Medical Research Council Cognitive Function and Ageing Study data

We demonstrate the method by using data from the MRC CFAS, adjusting all transition intensities for age (*A*), gender (*G*), years of education (*E*) and smoking status at the age of 60 years (*S*). We additionally adjust the intensity for the transition from state 2 to state 3 for the time spent in state 2. More specifically, we fit the model defined by Equations 1 and 2 for **Z**(*A*) = (1,*A*,*G*,*E*,*S*)^T^. The time-varying covariates in this model are age (*A*) and the time spent in state 2 (*W* − *A*), where *W* is age at the time of transition to state 2. We assume individuals who had a stroke before the beginning of the study to have been healthy at the age of 40 years (*A*_0_ = 40).

We undertake all the calculations for the likelihood contributions in C++ and perform the maximisation of the likelihood in R by using optim with the BFGS quasi-Newton optimising method 36–40. We set the resolution for the piecewise-constant approach to 0.25 year (3 months). We calculate the integrals for the calculation of the likelihood by using the composite Simpson's rule of numerical integration 35, with resolution equal to 0.05 year (18 days). We base the confidence intervals for the parameters on 450 bootstrap samples obtained using the studentised non-parametric bootstrap, as described by Davison and Hinkley 33. This involves resampling sampling units (i.e. individuals) from the data, stratifying by the following strata: (i) patterns A–D and (ii) patterns E and F. This stratification guarantees the same proportion of missing information with regard to the age at which individuals in patterns E and F had a stroke and guarantees the same amount of left truncation in all bootstrap samples. Failing to control for either or both, censoring and truncation, may introduce bias in the uncertainty of estimated parameters. To improve the accuracy of the studentised bootstrap method, the variance of the parameter estimates is stabilised by transforming the parameter scale by using the Box–Cox transformation 41.

Table 3 shows the results of the model. The bootstrap confidence intervals indicate that the time spent in state 2, the history variable, is not found to be statistically significant (95% confidence interval for *γ* includes 0). Although *γ* has a negative sign, which is the opposite than what would be expected according to the literature 24,1 (as survival has been shown to be adversely affected by the age at stroke and the risk of dying has been reported to be increased in the first several months after stroke 42,43), its absolute value is very small, and the confidence interval is relatively wide around zero. The positive coefficients for age reflect that having adjusted for gender, smoking, years of education and time spend in state 2; older people have increased mortality and hazard of having a stroke. The positive coefficients for gender reflect that men (as compared with women) have increased mortality at all ages and hazard for a stroke independently of their years of education or smoking status. These findings are consistent with those obtained by others 44,45. The positive coefficients for smoking show that smokers have increased mortality, as expected. Finally, higher education decreases the risk of dying on healthy individuals.

**Table 3:** Results of the model defined by Equations 1 and 2 for Z(*A*) = (1,*A*,*G*,*E*,*S*)^T^ fitted on the Medical Research Council Cognitive Function and Ageing Study data.

Covariate	Regression coefficient	Estimate	95% CI
Model intercepts	*β*_0.12_	− 8.184	( − 12.028, − 5.245)
	*β*_0.13_	− 11.150	( − 12.006, − 10.106)
	*β*_0.23_	− 7.472	( − 10.168, − 5.265)
Age (years)	*β*_*A*.12_	0.051	(0.013, 0.104)
	*β*_*A*.13_	0.101	(0.089, 0.111)
	*β*_*A*.23_	0.065	(0.039, 0.097)
Gender (men versus women)	*β*_*G*.12_	0.340	( − 0.050, 0.744)
	*β*_*G*.13_	0.298	(0.159, 0.427)
	*β*_*G*.23_	0.412	(0.128, 0.707)
Education (10 years or more)	*β*_*E*.12_	− 0.025	( − 0.472, 0.355)
	*β*_*E*.13_	− 0.228	( − 0.381, − 0.077)
	*β*_*E*.23_	0.159	( − 0.144, 0.507)
Smoking (current versus never/ex)	*β*_*S*.12_	0.203	( − 0.155, 0.591)
	*β*_*S*.13_	0.503	(0.383, 0.644)
	*β*_*S*.23_	0.347	(0.124, 0.647)
Time spent in state 2 (years)	*γ*	− 0.001	( − 0.021, 0.021)

*A* = age, *G* = gender, *E* = education, *S* = smoking. We assume transition intensities *q*_*ij*_ (*i*,*j*) ∈ {(1,2),(1,3),(2,3)} to be piecewise constant and introduce history in the process by fitting the time spent in state 2 as a time dependent covariate in *q*_23_. We based the confidence intervals on 450 bootstrap samples.

We assess the goodness of fit by using graphical methods by comparing observed with fitted survival curves and observed with expected prevalence in each state over time. When the model includes time-varying covariates, graphical illustration is not straightforward. We simulated a number of trajectories at an individual level under the fitted model to reflect the range of survival curves expected under the fitted model. The method of simulating trajectories at an individual level, known as microsimulation, has been used for studying the paths of dynamic processes in medical research, including studies of disability in the older population 46,47.

**Figure 5:**
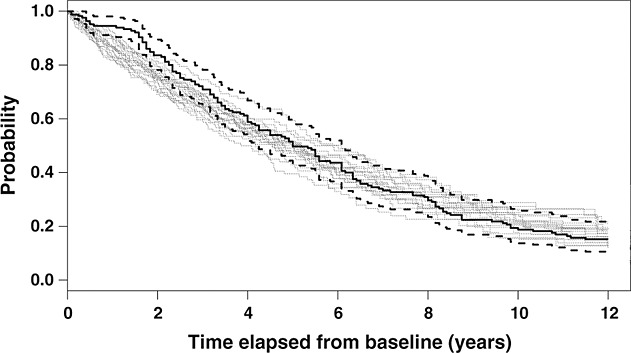
Probability of remaining in state 2 for individuals in patterns E and F. Solid lines correspond to the observed survival probability. Dotted lines correspond to simulated.

Figure 5 illustrates survival probabilities for individuals who had a stroke before the beginning of the study. The bold lines correspond to the Kaplan–Meier survival curve and its 95% confidence limits for the observed data in the MRC CFAS. The dotted lines correspond to the Kaplan–Meier survival curves obtained as follows:

1. We assume that there are 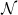 data sets, identical to the MRC CFAS data set at baseline, that is, data sets that include the same number of individuals with the same covariate specifications at baseline as the individuals in the MRC CFAS.
2. For every data set, we simulate one trajectory for every individual over time, assuming that the coefficients from Table 3 are correct. This generates 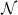 simulated data sets.
3. We plot the Kaplan–Meier survival curve for individuals in patterns E and F for all 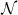 simulated data sets.

**Figure 6:**
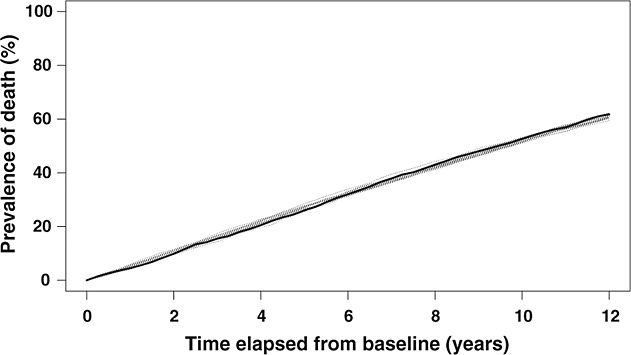
Prevalence of individuals in state 3. Solid lines correspond to the observed survival prevalence. Dotted lines correspond to simulated.

For individuals in patterns E and F, the model underestimates survival in the first 4 years, but model fit improves thereafter. Prevalence plots can be constructed for all states. However, the assessment of model fit by using prevalence plots becomes difficult because of the presence of interval censoring. Individuals are observed in state 2 later than when they actually move to state 2; thus, the observed prevalence in state 1 is greater than expected. The percentage of individuals in state 3 is not influenced by censoring, because exact times of deaths are available. The model predicts prevalence in state 3 remarkably well, considering the effect of the study design and the fact that the period *A*_*b*_ − *A*_0_ is on average 36 years for individuals in patterns E and F. Nevertheless, Figure 5 and Figure 6 can only show that some aspects of the model fit the data well. No claim about the overall goodness of fit can be made as graphical methods are not formal statistical tests. Titman and Sharples 48 discuss model diagnostics for multi-state models and present both graphical methods and formal statistical tests, but their discussion does not include our model.

## 7 Discussion

This paper presented a three-state illness–death model with no recovery in the presence of all types of censoring, where intensities for the transition from one state to another were allowed to change in a piecewise-constant manner. We handled interval censoring by using integration and handled left censoring by using an EM-inspired algorithm. We illustrated the method by using data for stroke from the MRC CFAS. The illness state was ‘history of stroke’, and we included time since having a stroke as a covariate in the intensity for the transition from the illness state to death.

We included individuals who had a stroke before the beginning of the study in the model after making the assumption that they were healthy at age *A*_0_ = 40. According to data published in ‘Key health statistics from general practice’ reports of the Office for National Statistics 30, this was a realistic assumption with regard to the age at which these individuals could be assumed to have been healthy in the past. Of those who had had a stroke and reported their history, 59% and 79% had had their first stroke within 5 and 10 years of baseline interview, respectively, therefore indicating that assuming a healthy outcome at the age of 40 years was a good estimate, and 98% expressed their first stroke after this age.

After choosing an appropriate value for *A*_0_, one way to handle left censoring is to include an extra observation for *all* individuals (not only those who had a stroke before the initiation of the study), in which everyone is in state 1 at age *A*_0_. Hence, left censoring can be treated as interval censoring by integrating out all possible transition times. When the time interval between age at baseline, *A*_*b*_, and *A*_0_ is disproportionately large as compared with the period of study follow-up, this approach may introduce considerable bias. For the MRC CFAS data, the assumption that all individuals were healthy at age *A*_0_ = 40 would add an extra 35 years of healthy life to the vast majority of individuals (2151 out of a total of 2316 individuals). Unobserved person-years for the period between *A*_0_ and *A*_*b*_ would be treated as observed in the calculation of the likelihood. If the study had recruited individuals from age *A*_0_, some of them would have died within [*A*_0_,*A*_*b*_]. Thus, their data would have been left truncated. Left truncation makes the estimation of the left-censored entry time in state 2 more difficult. Including person-years for the period [*A*_0_,*A*_*b*_] in the likelihood, failing to include individuals whose data would have been observed within that period, or otherwise adjust for left truncation may introduce bias.

The proposed method overcomes this problem by imputing the sojourn time in state 2 for individuals who had a stroke before baseline and by utilising an EM-inspired algorithm. All the formulas for the likelihood contributions are conditional on state at the beginning of the study and bypass the problem of left truncation. We calculate the likelihood over the period of study follow-up, [*A*_*b*_,*A*_*N*_], and the assumption that study participants were healthy at age *A*_0_ is made only for those who were observed in state 2 at baseline. In particular, for these individuals, we assume that the underlying stochastic process that governs transitions between states is the same throughout the period [*A*_0_,max{*A*_*N*_}], where max{*A*_*N*_}is the maximum age *A*_*N*_ observed in the data. This involves the extrapolation of the formulas for the transition intensities back in time to age *A*_0_. This assumption offers a practical solution to an otherwise unsolvable problem. Nevertheless, the lack of information over the period from age *A*_0_ to *A*_*b*_ makes this assumption unverifiable. The introduced EM-inspired algorithm is an intuitively appealing method because missing values of age of transition to state 2 are imputed under a specified model iteratively until they converge to optimum values that maximise the likelihood of the complete data. Although, in simulation studies no problems have been experienced in the implementation of the method and the method has been shown to be able to produce unbiased estimates, the mathematical properties of the proposed EM-inspired algorithm regarding convergence and susceptibility to bias require further investigation.

A simulation study showed that the performance of the method is affected by the choice of *A*_0_. The performance of the method improves as *A*_*b*_ − *A*_0_ decreases. The method was found to perform well when the data are subject to moderate left censoring, where moderate left censoring is with respect to both the average length of [*A*_0_,*A*_*b*_] and the percentage of left-censored data. Data subject to moderate left censoring are common in medical research. For example, when modelling HIV, the difference between age at baseline and age at which all individuals could be assumed to have been healthy would commonly be less than 10 years.

Fitting the model defined by Equations 1 and 2 by using piecewise-constant transition intensities and age as the time scale enables the explanatory variables *X*_1_,*X*_2_, …, *X*_*r*_ to vary with age *A*. When the change of a covariate *X*_*s*_ for *s* = 1, …, *r* over time cannot be calculated using age in a deterministic way, the trajectory of *X*_*s*_ needs to be estimated. This can be achieved by carrying forward the last observed value of *X*_*s*_ or by jointly modelling the trajectory of *X*_*s*_ with the transition intensities (Equations 1 and 2). For example, the time since the beginning of the study can be obtained deterministically by the difference in age between two time points, whereas the trajectory of a covariate such as an individual's blood pressure over time cannot. One way of estimating individuals' blood pressure is by carrying forward the last observation. In this way, time-varying covariates remain constant between successive follow-up visits and at any time they are equal to the value obtained from the latest follow-up visit. To avoid having follow-up visits with updated information on covariates lying within any subinterval where transition intensities are assumed to be constant, all study follow-up visits can be included as time points in the age grid of the piecewise-constant approach. Another way is to predict individuals' blood pressure by using linear regression adjusting for possible confounders. Depending on the application, more complex methods of prediction may be appropriate.

The piecewise-constant approach is to be distinguished from a discretization used to approximate the integrals in the likelihood contributions. In fact, the proposed method involves two time grids. The first grid is used for numerical integration and can be chosen to have a fine resolution. The second time grid corresponds to the intervals within which transition intensities are assumed to remain constant (piecewise-constant hazards). For a given time of onset of state 2, the integrand of a likelihood contribution is being calculated using the piecewise-constant approach described in Section 3.2. Thus, for any two different times of onset of state 2, the integrand is calculated in a different way. The reason all possible transition times are integrated out is because the time of onset of state 2 is unknown.

When covariates *X*_1_,*X*_2_, …, *X*_*r*_ do not depend on age, the only time-varying covariates are age, *A*, and time spent in state 2, *A* − *W*. In this case, the model defined by Equations 1 and 2 can be fit without adopting a piecewise-constant approach for the transition intensities. Although the piecewise-constant approach was not necessary in the application presented in this paper, we fitted the model defined by Equations 1 and 2 by using piecewise-constant transition intensities because this approach is more general and allows the inclusion of time-varying covariates other than age and time spent in state 2. When such covariates are included in the model and a piecewise-constant approach is adopted, we believe that expressing the model in the form of Equations 1 and 2) provides better intuition and is more concise as compared with the form of the product of a baseline intensity function and a factor that includes the effects of covariates.

Applying the method to the MRC CFAS data (Newcastle centre) showed that the time since having a stroke was not statistically significant. Age was shown to increase mortality and the risk of having a stroke. Compared with women, men were found to have increased mortality at all ages and independently of their years of education or smoking status. These findings are consistent with those obtained by others 44,45. Smoking was shown to increase mortality, whereas higher education was shown to decrease the hazard of death on healthy individuals. Nevertheless, whether education alone can affect the risk of dying needs further discussion. Levels of education are usually associated with socio-economic status and other causal factors, such as quality of health care received. Although the MRC CFAS data were subject to left censoring, graphical assessment of model fit showed that the model fitted some aspects of the data very well. Nevertheless, graphic assessment of model fit in multi-state modelling cannot assess the overall goodness of fit, even when the model is correctly specified. The presence of interval censoring may cause discrepancies between the observed and expected prevalence in some states of the multi-state model. Individuals are observed in state 2 later than the time they move to state 2. Hence, observed prevalence in state 1 is greater than the expected, even when the model is correctly specified and fits the data very well. Graphs can show that some aspects of the model fit the data well. Titman and Sharples 48 have developed formal statistical tests for diagnosing model fit in multi-state modelling. However, these methods fall beyond the scope of this study.

This paper has presented a method to fit semi-Markov models in the presence of all types of censoring and left truncation. The method allows the inclusion of time-varying covariates that may change over time in a stochastic way and can be applied successfully in a range of medical applications with data subject to moderate left censoring. The parametric form of the piecewise-constant approach presented in this paper offers flexibility and, additionally, the option to use the fitted model to extrapolate into the future (prediction). This is particularly important in research areas such as the estimation of life expectancies. Furthermore, the parametric form of the transition intensities facilitates the adjustment for left censoring. Finally, this paper has proposed a new graphical way of investigating goodness of fit when the model includes time-varying covariates, which is based on microsimulation.
